# Identification of Featured Metabolism-Related Genes in Patients with Acute Myocardial Infarction

**DOI:** 10.1155/2020/8880004

**Published:** 2020-11-28

**Authors:** Hang Xie, Enfa Zha, Yushun Zhang

**Affiliations:** Department of Structural Heart Disease, The First Affiliated Hospital of Xi'an Jiaotong University, Xi'an 710061, China

## Abstract

**Objective:**

A growing body of emerging evidence indicates that metabolic processes play a pivotal role in the biological processes underlying acute myocardial infarction (AMI). The aim of the current study was to identify featured metabolism-related genes in patients with AMI using a support vector machine (SVM) and to further explore the value of these genes in the diagnosis of AMI.

**Methods:**

Gene microarray expression data related to AMI were downloaded from the GSE66360 dataset in the Gene Expression Omnibus (GEO) database. This data set consisted of 50 AMI samples and 49 normal controls that were randomly classified into a discovery cohort (21 AMI samples and 22 normal controls) and a validation cohort (28 AMI and 28 normal controls). We applied a machine learning method that combined SVM with recursive feature elimination (RFE) to discriminate AMI patients from normal controls. Based on this, an SVM classifier was constructed. Receiver operating characteristic (ROC) analysis was used to investigate the predictive value for the early diagnosis of AMI in the two cohorts and was then further verified in an independent external cohort.

**Results:**

Three metabolism-related genes were identified based on SVM-RFE (*AKR1C3*, *GLUL*, and *PDE4B*). The SVM classifier based on the three genes allowed for excellent discrimination between AMI and healthy samples in both the discovery cohort (AUC = 0.989) and the validation cohort (AUC = 0.964), and this was further confirmed in the GSE68060 dataset (AUC = 0.839). Additionally, the SVM classifier allowed for perfect discrimination between recurrent AMI events and nonrecurrent events in the GSE68060 cohort (AUC = 0.992). GO and KEGG pathway enrichment analysis of the identified featured genes revealed significant enrichment of specific metabolic pathways.

**Conclusion:**

The identified metabolism-related genes may play important roles in the development of AMI and may represent diagnostic and therapeutic biomarkers of AMI.

## 1. Introduction

AMI results from interrupted blood flow to a certain area of the heart and is considered one of the primary causes of disability and death from cardiovascular disease worldwide, thus posing a serious threat to human health [1]. Over the last decade, the primary therapeutic strategies, including percutaneous coronary intervention, coronary artery bypass surgery, and medications, have improved the prognosis of AMI. However, approximately one-third of eligible patients failed to receive early reperfusion therapy due to late detection [2]. Early diagnosis and interventional therapy are beneficial in that they significantly reduce mortality and improve prognosis [3]. Therefore, an early diagnosis may markedly contribute to overall survival.

The diagnosis of AMI is typically based upon the observation of changes in a surface electrocardiogram (ECG) and blood levels of sensitive and specific biomarkers such as cTnI/T and CKMB. However, the sensitivity and specificity of these biomarkers remain unsatisfactory, often resulting in a lack of diagnosis or misdiagnosis [3, 4]. Based on this, potential biomarkers possessing high sensitivity and specificity for early diagnosis of AMI are urgently required and could ultimately contribute to improved clinical survival. Gene expression profiles related to AMI have been previously studied. The differentially expressed genes related to cardiovascular events exhibit similar variation components to those of AMI-related genes. Regardless of if they are upregulated or downregulated, they change in the same direction [5]. This study suggests that differentially expressed genes may provide a new biomarker for predicting AMI. Recently, metabolic pathways in cardiovascular disease have been demonstrated to represent potential new valuable targets for drug therapy [6, 7]. A recent study revealed that exercise improves cardiac function and glucose metabolism in mice with experimental myocardial infarction by inhibiting phosphorylated histone deacetylase 4 (*HDAC4*) and upregulating glucose transporter 1 (*GLUT1*) expression. These results demonstrated that metabolic processes play a pivotal role in the biological processes underlying AMI. Metabolism-related genes have been studied in the context of cardiac ischemia. By inhibiting miR-92a-3p, LNA-92a can increase endothelial cell autophagy and regulate the expression of metabolism-related genes, thereby increasing myocardial fatty acid uptake and mitochondrial function. These prosurvival mechanisms may reduce tissue damage after myocardial infarction [8]. However, the role of metabolism-related genes in AMI remains to be fully elucidated.

In this study, differentially expressed metabolism-related genes were identified in normal and AMI samples. Next, an SVM classifier consisting of three risk genes was established. This classifier allowed patient samples to be distinguished from normal controls.

## 2. Materials and Methods

### 2.1. Microarray Data

To investigate metabolism-related genes, the microarray data for AMI were collected from the GEO (http://www.ncbi.nlm.nih.gov/geo/) database under the accession number GSE66360, where 50 AMI samples and 49 normal controls were included. The dataset was divided into a discovery cohort (21 AMI samples and 22 normal controls) and a validation cohort (28 AMI samples and 28 normal controls). Furthermore, the GSE48060 dataset consisting of 31 AMI patients and 21 healthy controls was obtained to confirm the performance of the SVM classifier. Additionally, 5 recurrence and 26 no-recurrence samples over a 1.5-year follow-up period were included in the GSE48060 dataset. All datasets were produced using the Affymetrix Human Genome U133 Plus 2.0 Array. Background correction and normalization were performed using linear models for the microarray data (LIMMA) software package. Normalization between arrays was performed using the quantile algorithm in LIMMA.

### 2.2. Metabolism-Related Genes

Metabolism-related genes were obtained from the Molecular Signatures Database v7.1 (MSigDB) (http://software.broadinstitute.org/gsea/msigdb) by searching using the term “metabolism.” The C2 (c2.cp.kegg.v7.1.symbols.gmt) subcollection was selected as the reference gene set (Supplementary Table 1). A total of 948 unique metabolism-related genes were obtained. Next, we extracted the metabolism-related gene expression matrix from the GSE66360 dataset using the R language merge package. Finally, a gene expression matrix consisting of 862 gene expression values was obtained in the discovery cohort.

### 2.3. Screening of Differentially Expressed Metabolism-Related Genes

The analysis of differentially expressed metabolism-related genes between AMI and normal samples was conducted using the LIMMA package implemented in the R statistical package (http://www.r-project.org). The threshold for the identification of differentially expressed genes was set at a *P* value of <0.05 and a ∣log2fold change (FC)  | ≥1.

### 2.4. Featured Gene Selection and the SVM Classifier Construction

The featured selection technique is an efficient tool for identifying meaningful information from a given gene dataset [9]. SVM is a supervised learning model that is aimed at classifying data points by maximizing the distance of a hyperplane for classification and regression analysis with high accuracy [10]. SVM-RFE is a popular feature selection technique and has exhibited promising and expanding applications for the analysis of high-dimensional data. It is much more robust with regard to data overfitting and classification accuracy than many other feature selection methods, and this technique has demonstrated its power in many fields, including metabolomics [11–13]. Therefore, we applied a machine learning method that combined SVM with RFE to select the best parameters for gene selection among all differentially expressed metabolism-related genes. Using this algorithm, optional feature genes were identified as risk genes in the context of AMI. Next, the identified feature genes were added into an SVM classifier with a radial basis function (RBF) kernel and 5-fold cross-validation to achieve predictions. To test the value of the identified featured genes, a heat map was clustered using the pheatmap package in R for all samples in the two cohorts (clustering method = ^“^ward^”^). The Euclidean distance was used to cluster samples. Furthermore, the discriminatory power of the SVM classifier was measured according to the AUC (defined as the area under the receiver operating curve) in both cohorts, and this was further validated in the independent external cohort. Additionally, the performance of the SVM classifier was further explored in terms of AMI recurrence and nonrecurrence.

### 2.5. Functional Enrichment Analysis of Identified Feature Genes

To explore the functions and pathways of the identified feature genes, gene ontology (GO) and Kyoto Encyclopedia of Genes and Genomes (KEGG) pathway enrichment analyses were performed to identify potential functional components and pathways underlying numerous genes using the clusterProfiler package [14]. *P* < 0.05 was considered to be statistically significant.

### 2.6. Statistical Analysis

The differentially expressed metabolism-related genes were identified using the Limma package with *P* < 0.05 and ∣log2fold change (FC)  | ≥1 as the cut-off criteria. Featured gene selection was performed using the RFE function in the caret package with 5-fold cross-validation. The SVM classifier was constructed using R package e1071 with 5-fold cross-validation. Hierarchical clustering analysis was used for the identified featured genes using the pheatmap package in R. ROC analysis was performed, and the area under the curve (AUC) was calculated to evaluate the predictive performance of the classifier. *P* < 0.05 was considered to indicate a statistically significant difference. All statistical analyses were performed using R software (version 3.6.3, http://www.r-project.org).

## 3. Results

### 3.1. Identification of Differentially Expressed Metabolism-Related Genes and Feature Genes

**Figure 1:**
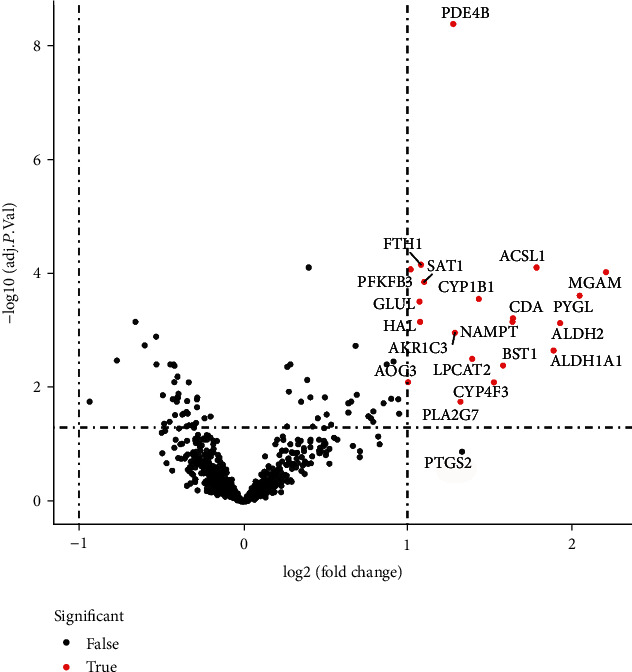
Differential expression of metabolic-related genes in AMI tissue and normal samples.

**Figure 2:**
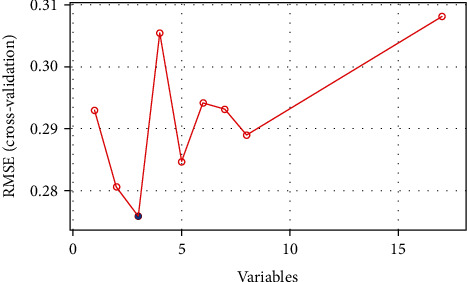
A plot of feature metabolic-related gene selection by recursive feature elimination.

**Figure 3:**
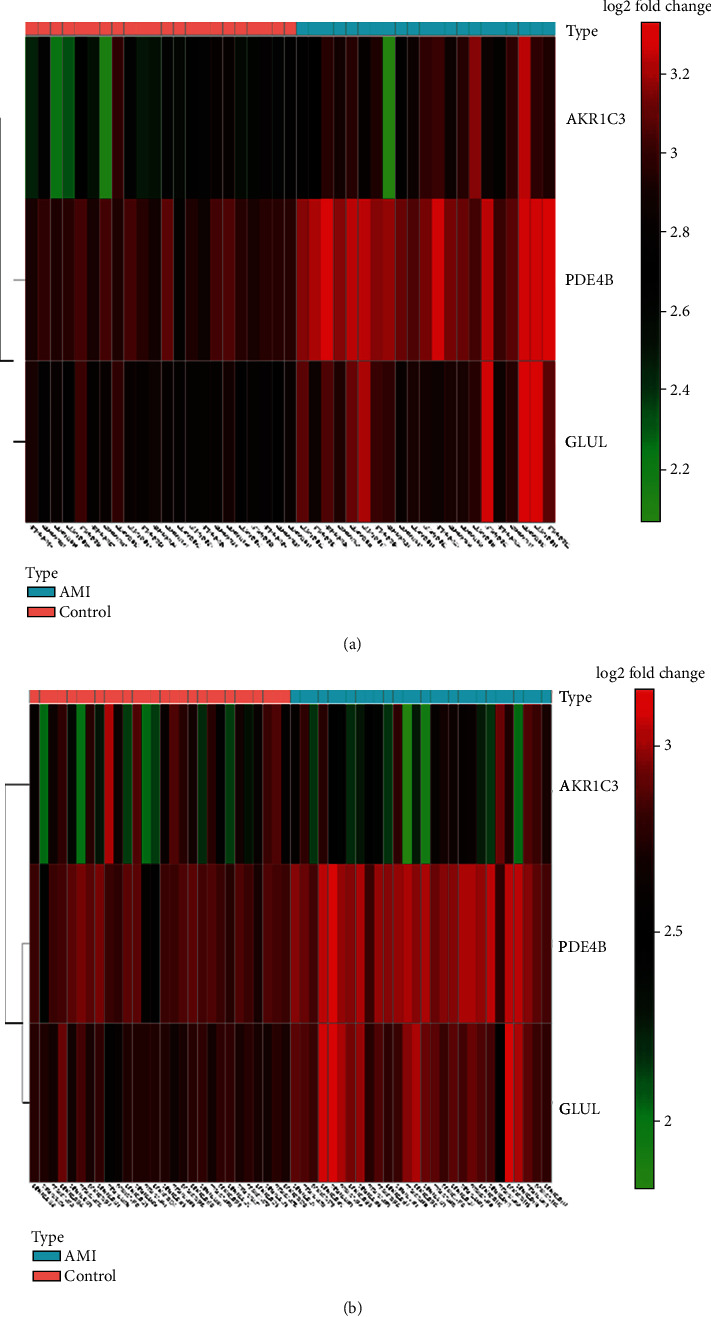
Hierarchical clustering analysis demonstrates identified metabolic-related gene expression patterns between AMI and normal tissues in the discovery cohort (a) and validation cohort (b).

**Figure 4:**
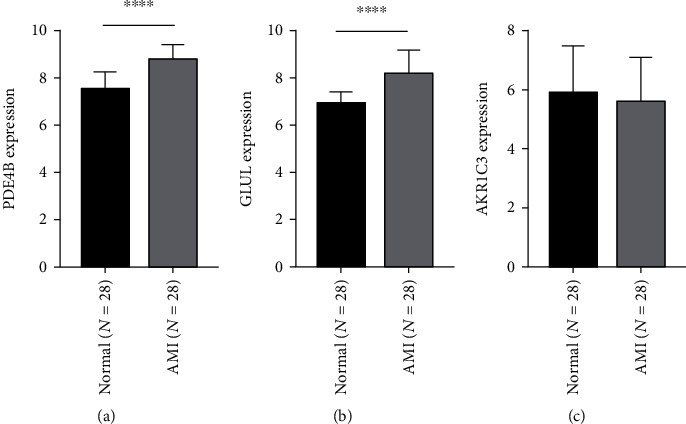
Validation of expression level of identified feature genes in patients with AMI and normal tissues in the validation cohort. (a) PDE4B, (b) GLUL, and (c) AKR1C3.

A total of 17 differentially expressed upregulated genes were identified between AMI tissues and normal tissues (Figure 1.). Based on the SVM-RFE algorithm, three genes (*AKR1C3*, *GLUL*, and *PDE4B*) with minimum root mean square error were fit into the SVM classifier (Figure 2). Hierarchical clustering analysis in the discovery cohort (Figure 3(a)) and the validation cohort (Figure 3(b)) revealed that patients could be clearly separated into two clusters based on the expression levels of the three identified feature genes. To validate the expression levels of three featured genes, the identified featured genes were confirmed in the validation cohort. As shown in Figure 4, the expression levels of two featured genes (*GLUL* and *PDE4B*) in AMI tissues were significantly higher than those in the control group (*P* < 0.05).

### 3.2. Diagnostic Value of Three Feature Genes in AMI

**Figure 5:**
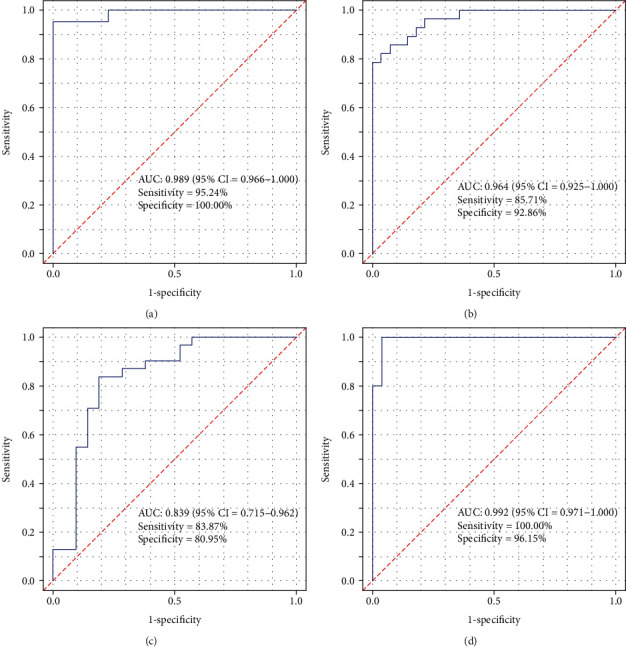
Receiver operating characteristic curves of support vector machine classifier for the discovery cohort (a), the validation cohort (b), the independent external cohort (c), and ROC analysis of the SVM classifier for AMI recurrent prediction in the independent external cohort (d).

As presented in Figure 5(a), the results of the 5-fold cross-validation illustrated that the SVM classifier allowed for good classification in the discovery cohort between AMI and normal controls with an AUC of 0.989 (95% CI = 0.966-1.00), a sensitivity of 95.24%, and a specificity of 100.00%. The SVM classifier demonstrated excellent discriminatory ability in the validation cohort with an AUC of 0.964 (95% CI = 0.925-1.000), a sensitivity of 85.71%, and a specificity of 92.86% (Figure 5(b)). The discrimination power was confirmed in the independent GSE48060 cohort with an AUC of 0.839 (95% CI = 0.715-0.962), a sensitivity of 83.87%, and a specificity of 90.95% (Figure 5(c)). Furthermore, we investigated the discrimination ability of the classifier in the context of recurrent AMI. The classifier exhibited outstanding discrimination ability of recurrent AMI with an AUC of 0.992 (95% CI = 0.971-1.00), a sensitivity of 100%, and a specificity of 96.15% (Figure 5(d)).

### 3.3. Functional Analysis of Feature Genes

**Figure 6:**
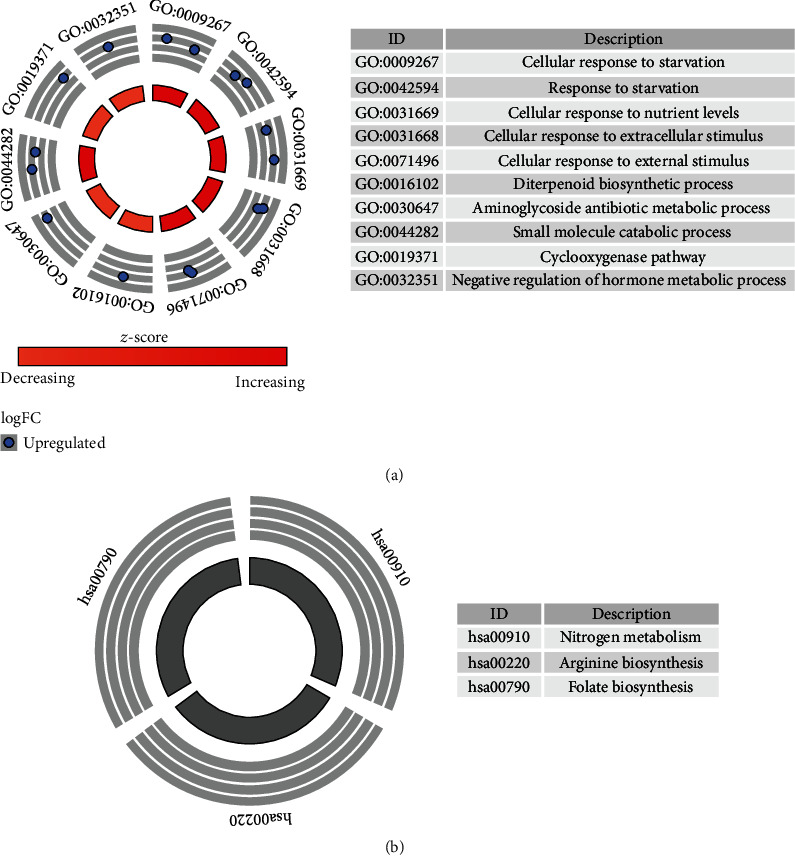
GO and KEGG pathway analysis of identified feature genes.

Based on our results, cellular response to starvation, cellular response to nutrient levels, cellular response to extracellular stimulus, diterpenoid biosynthetic process, small molecule catabolic process, cyclooxygenase pathway, and negative regulation of hormone metabolic processes were the most significantly enriched biological processes (Figure 6(a)). Additionally, nitrogen metabolism, arginine biosynthesis, and folate biosynthesis were considered to be the most remarkably enriched pathways (Figure 6(b)).

## 4. Discussion

AMI remains a primary cause of death and disability worldwide despite significant improvements in diagnosis. The in-hospital mortality for AMI remains high [15]. Recently, metabolism-related processes have been reported to be involved in the pathogenesis of AMI [7, 16]. *PRODH* overexpression increases the number of gene transcripts related to metabolism, and this gene is related to the maintenance of normal mitochondrial function, ATP level, and redox homeostasis of human cardiomyocytes in a hypoxic environment [17]. However, the potential role of metabolism-related genes in AMI remains poorly understood. Single-nucleotide polymorphisms (SNPs) in some lipid metabolism-related genes are closely related to blood lipids and can cause coronary artery disease [18]. A study from Pakistan revealed that SNPs in lipid metabolism genes are significantly associated with MI susceptibility [19]. Circadian rhythm disorders can cause worsening of atherosclerosis [20]. A large number of metabolism-related genes exhibit a circadian rhythm [21]. Zhu et al. found that abnormal light can aggravate the circadian rhythm of lipid metabolism genes [22]. The above studies indicate that metabolic genes may increase the risk of AMI by affecting lipid metabolism.

To identify the metabolism-related genes that are involved in AMI, GSE66360 datasets were used to screen differentially expressed genes in patient tissues and control tissues. By comparing the expression levels of metabolism-related genes between AMI patients and healthy samples, we found that 17 genes were differentially expressed in AMI compared to healthy samples, indicating that metabolism-related genes may play critical roles in the occurrence of AMI. Next, three featured genes in AMI samples were identified using the SVM-RFE algorithm that allows AMI samples to be distinguished from normal samples. The SVM classifier based on the identified featured genes allowed for good classification with an AUC of 0.989 for the patient samples. The discrimination power values of the classifiers for the validation cohort and the independent validation cohort were 0.964 and 0.839, respectively. Furthermore, the SVM classifier can successfully distinguish patients with recurrent and nonrecurrent AMI with an AUC of 0.992. Therefore, the present study suggested that the featured genes could provide useful markers for identifying patients with AMI.

The present study demonstrated the potential value of metabolic-related genes in the context of AMI in the clinical setting. *GLUL*, *PDE4B*, and *AKR1C3* were identified as potential metabolism-related genes that were associated with AMI and the recurrence of AMI. Glutamate-ammonia ligase (*GLUL*) belongs to the glutamine synthetase family and functions to catalyze the synthesis of glutamine from glutamate and ammonia in an ATP-dependent reaction [23]. Genetic studies have revealed a *GLUL* rs10911021 polymorphism that is associated with cardiovascular disease morbidity and mortality among people with type 2 diabetes [24]. Genome-wide association analyses suggested that the *GLUL* may regulate the risk of coronary heart disease by affecting glutamate/glutamine metabolism and the activity of the *γ*-glutamine cycle [25]. Coronary heart disease is the primary cause of death in patients with diabetes, and genetic factors can also act as risk factors for increased mortality. Clinical studies from European populations indicate that SNP rs10911021 is an independent risk factor for all-cause mortality in patients with type 2 diabetes, and the risk may be due to cardiovascular disease [26]. *GLUL* has also been reported to be involved in endothelial cell motility, a process that affects endothelial cell junctional integrity [27]. These studies provide possible explanations for the role of *GULU* in AMI.

The phosphodiesterase 4B (*PDE4B*) gene is a member of the type IV cAMP-specific, cyclic nucleotide phosphodiesterase (PDE) family that regulates the cellular concentrations of cyclic nucleotides and thereby plays a role in signal transduction. In the myocardium, the PDE3 and PDE4 families are primarily used to degrade cAMP and regulate excitation-contraction coupling (ECC). PDE4 is responsible for 40% of cAMP-hydrolysis activity in human heart tissue [28]. Animal experiments have demonstrated that the primary function of PDE4 is the control of the cAMP signal, and PDE4 is responsible for the majority of the cAMP degradation activity in rat ventricular cells [29, 30]. In the heart, cAMP regulates contraction, relaxation, and autonomy. When the regulation of this molecule is imbalanced, it significantly promotes the development of heart disease. Wang et al. demonstrated that a moderate increase in cAMP levels prevents the Ca2+-induced mitochondrial permeability transition pore (MPTP) from opening through Epac1, thus affecting the death of cardiomyocytes [31]. A number of studies have also confirmed that PDE4 is related to arrhythmia and heart failure [32–34]. These studies may reveal the molecular mechanism of *PDE4B* in AMI; however, more detailed mechanisms require further exploration.

The aldo-keto reductase family 1 member C3 (*AKR1C3*) gene is the only 17*β*-HSD that is not a short-chain dehydrogenase/reductase, and this gene encodes a member of the aldose/ketoreductase superfamily. *AKR1C3* has been confirmed to play a regulatory role in a variety of endocrine diseases [35]. Su et al. confirmed that *AKR1C3* modulates vasodilation and vasoconstriction by regulating the biosynthesis of prostaglandins [36]. Prostacyclin is a vasodilator that can inhibit platelet activation. Imbalances in prostacyclin production can result in an increased risk for coronary events [37]. *AKR1C3* did not show a significant difference in expression in AMI and control in the validation dataset GSE48060. Based on this, *AKR1C3* may influence AMI; however, given the small number of research studies examining this process, more research is required to provide a fuller understanding of the relationship between *AKR1C3* and AMI.

As revealed in the GO and KEGG pathway analyses, these featured genes were primarily enriched in metabolism-related processes, thus indicating that metabolism-related genes play an important role in AMI. As an important process of lipid metabolism, autophagy is activated by starvation [38, 39]. Autophagy is also a regulated pathway of cellular deprivation [40]. Prostaglandins have been reported to be related to AMI [41, 42]. Thyroid hormone exerts an important therapeutic effect by reducing the infarct size and improving myocardial function after acute myocardial infarction [43]. Clinical studies have shown that in adults with hypertension, both low folate and high folate levels are associated with an increased risk for death from cardiovascular disease [44]. Arginine has also been confirmed to be associated with AMI [45, 46].

Our study does possess certain limitations. First, we failed to validate the discriminatory ability of the SVM classifier in the independent cohort with respect to the recurrent event. Second, although the ROC analysis of the support vector machine classifier for AMI recurrence prediction yields good results, the sample size for recurrent AMI was small, and its accuracy requires further verification using larger sample sizes. Finally, it should be noted that this research was based on bioinformatics analyses. Therefore, further validations *in vivo* and *in vitro* are required.

## 5. Conclusion

The present study identified three metabolic-related genes (*GLUL*, *PDE4B*, and *AKR1C3*) in patients with AMI, and these genes may be useful as potential biomarkers in the diagnosis of AMI. Knowledge of these genes will improve our understanding of the molecular mechanism underlying the occurrence of AMI.

## Data Availability

The raw data of this study are derived from the GEO data portal (https://www.ncbi.nlm.nih.gov/geo/; accession numbers: GSE66360 and GSE68060), which is a publicly available database.

## Supplementary Materials

Supplementary Materials Supplementary table 1: reference gene set.
